# The Age Distribution among Children Seeking Medical Treatment for Precocious Puberty in Taiwan

**DOI:** 10.3390/ijerph17186765

**Published:** 2020-09-17

**Authors:** Pen-Hua Su, Jing-Yang Huang, Cho-Shun Li, Hua-Pin Chang

**Affiliations:** 1 School of Medicine, Chung Shan Medical University, Taichung 41354, Taiwan; ninaphsu@gmail.com; 2 Department of Pediatrics, Chung Shan Medical University Hospital, Taichung 41354, Taiwan; 3 Center for Health Data Science, Chung Shan Medical University Hospital, Taichung 41354, Taiwan; wchinyang@gmail.com; 4 Institute of Medicine, Chung Shan Medical University, Taichung 41354, Taiwan; 5 Department of Neurosurgery, Chung Shan Medical University Hospital, Taichung 41354, Taiwan; hkli@ms28.hinet.net; 6 Department of Nursing, Asia University, Taichung 41354, Taiwan; 7 Department of Medical Research, China Medical University Hospital, China Medical University, Taichung 41354, Taiwan

**Keywords:** precocious puberty, children, seeking medical care, prevalence and incidence

## Abstract

Objective: Children with precocious puberty (PP) may have increased physiological and psychological problems. In this study, we aimed to explore the trend of parents seeking medical care for their children with precocious puberty. Methods: The Taiwan National Health Insurance Research Dataset (NHIRD) was used to estimate the prevalence (2000–2013) and incidence (2002–2013) of PP (ICD-9 code: 259.1) among boys aged 0–11 years and girls aged 0–10 years. The proportions of PP management within 1 year from the date of first diagnosis were also compared between two periods (2002–2007 and 2008–2012). The trends of PP prevalence or incidence were determined by join-point regression. Results: In 2000, 309 boys and 2706 girls had at least one visit for PP, the crude prevalence rates (per 10,000 persons) were 0.99 (95% confidence interval, 95% CI 0.87–1.14) and 13.56 (95% CI 13.01–14.13) in boys and girls, respectively. In 2013, the crude prevalence rates increased to 7.01 (95% CI 6.56–7.84) and 110.95 (95% CI 108.97–112.96) in boys and girls, respectively. A total of 2584 girls and 207 boys with incident PP cases were identified in 2002, and 7498 girls and 739 boys were identified in 2013. For girls, the incidence rates (per 10,000 person-years) were 16.17 (95% CI 15.55–16.80) and 70.23 (95% CI 68.65–71.83) in 2002 and 2013, respectively. For boys, the incidence rates were 1.09 (95% CI 0.95–1.24) and 5.72 (95% CI 5.32–6.15) in 2002 and 2013, respectively. The sex ratio (F:M) of the incidence of PP cases was 14.89 in 2002 and 12.28 in 2013. Conclusion: In this study, from 2000 to 2013, the frequency of visiting pediatric endocrinology outpatient clinics for precocious puberty increased in both genders. We advocate that it is important to pay increased attention to children’s health, environmental hormones, and diet. Researchers should consider how to survey precocious puberty and offer parents more education to avoid the waste of medical resources or delays in seeking medical care.

## 1. Introduction

Precocious puberty (PP) is defined as secondary sexual characteristics that occur before the age of 8 in girls or 9 in boys [1]. PP may be caused by central or peripheral mechanisms [2,3]. One of the causes of PP is the early activation of pulsatile gonadotropin-releasing hormone (GnRH) secretions (central precocious puberty, CPP) resulting from hypothalamic tumors, lesions, or genetic conditions, but most causes are unclear [1,4]. At least half of children with PP are not treated because their pubertal manifestations will regress or stop progressing. PP may affect children’s final body height and increase the risk of mental health problems [1,2,3,4].

Factors that increase a child’s risk of precocious puberty include gender (PP is more prevalent in girls), ethnicity, overweight, obesity, exposure to environmental toxins and pollutants, and extensive use of plastics and preservatives [1,2,3,4,5,6]. Over the last few decades, Taiwanese children’s dietary habits have been changing, particularly with regard to the intake of cakes, sweets, and sugary drinks. Excessive energy intake and a sedentary lifestyle in young people has led to the increase in obesity [7]. According to Chu’s survey, the prevalence of obesity in Taiwanese children (defined as body weight > 120% of the mean body weight with age and gender specification) was 12.4%, 14.8% and 15.6% among boys and 10.1%, 11.1%, and 12.9% among girls in 1980, 1986, and 1996, respectively. Chu also found that the prevalence of children’s obesity in Taiwan increased steadily from 1980 to 2000, in particular in boys [7,8,9].

With the help of U.S. aid agency loans, the Formosa Plastics Group was established in 1954 as Taiwan’s first polyvinyl chloride (better known as PVC) manufacturing facility [10]. Taiwan was then called a “petrochemical kingdom”. According to an Environmental Protection Administration report, more than 20% of the waste in landfills in Taiwan is plastic waste [10,11]. Taiwan’s plastic production continues to increase, and various phthalate esters (PAEs) are also widely added to the production of polyvinyl chloride (PVC), polyethylene, and polypropylene [10]. Plastic products, clothes, toys, medical equipment, cosmetics, and construction products (such as flooring, wallpapers, and automotive parts), all contain phthalate esters (PAEs), and the content of di (2-ethyl hexyl) phthalate (DEHP) in different products ranges from 20% to 40% [5,11]. Phthalate esters (PAEs) are combined physically rather than chemically with the polymer matrix, and so PAE compounds can be readily released into the atmosphere, water, soil, sediments, and foods in relatively large quantities due to their large-scale and worldwide use [5,10,11,12,13]. In Taiwan, the PAE pollution in rivers has reached a critical level that cannot be ignored [11,13,14,15].

Di-n-butyl phthalate (DnBP) and di (2-ethyl hexyl) phthalate (DEHP) are the two most frequently detected PAE additives, and both are included in the priority list produced by the US Environmental Protection Agency (USEPA) as potential endocrine disrupting compounds (EDCs) [13,14,15,16]. EDCs can act through several mechanisms at any level in the hypothalamic-pituitary-gonadal (HPG)-peripheral tissue endocrine axis. Directly, EDCs may affect genes or HPG pathways that are unique to puberty [16,17]. Indirectly, EDCs can act as obesogens and change the metabolic programming during fetal and early childhood development, resulting in alterations in the metabolic and peripheral hormones associated with the onset of puberty [5,17,18,19,20]. A Taiwan birth cohort study indicated that maternal exposure to phthalates might affect the sex steroid hormones status in the fetal and newborn stage [21]. In addition, Su et al. conducted a birth cohort study, in which evidence of the association between phthalate exposure and decreased uterine volume was found [22].

On 23 May 2011, all major news media in Taiwan reported the explosive news on the incidence of plasticizer-contaminated food on the market. One of the major newspapers even described the incidence in its headline as “the biggest plasticizer-contaminated food episode in human history” [23,24]. The contaminated food items, including beverages (juice, sport drinks, and tea), fruit jams/jelly, and dietary supplements, were taken off the shelves by mandate of the Department of Health [24,25]. With the widespread reports on the undesirable effects of plasticizer intake without the provision of the complete information regarding exposure time and period (dose and dose rate), the general public was very nervous about the possible aftermath of carcinogenesis and sex organ malformation in babies or young children. This incident resulted in tremendous social costs [24,25].

As stated above, Taiwan’s rapid industrialization led to changes in diet and lifestyle, and environmental pollution and food safety issues may affect the development of children’s reproductive functions. To our knowledge, four epidemiological studies have estimated the national incidence of PP [26,27,28,29]. The different rates of prevalence and incidence of PP reported from previous studies may be related to the diverse age of precocious puberty definitions, varying research methods, and racial diversity. Most articles focused solely on the time, prevalence, and incidence of precocious puberty. However, few studies paid attention to children who did not meet the criteria of precocious puberty (girls >8 years old and boys >9 years old). These children were typically delayed in seeking medical treatment. Taiwan established a system of universal National Health Insurance (NHI) in March 1995. Today, the NHI covers more than 99% of Taiwan’s population and provides enrollees with almost free access to healthcare with very modest co-payment at most clinics and hospitals. Therefore, this article utilizes the National Health Insurance Research Dataset (NHIRD) [30], which contains comprehensive information on diagnoses, testing, and clinical visits, essentially covering the entire population of Taiwan, to analyze the status of children of different ages seeking medical assistance.

## 2. Methods

### 2.1. Data Source

The Taiwan National Health Insurance Research Dataset (NHIRD) is retrieved from the National Health Insurance Program, which is a social single-payer system covering more than 99% of the Taiwanese population (23.37 million inhabitants in 2013). This study retrieved the 2000–2013 non-sampled NHIRD, including the registry for beneficiaries (the demographic variables, date of enrolment, date of dropout, and characteristics of group insurance applicants), administrative claims in outpatient visits (including the diagnosis of disease (International Classification of Disease; ICD-9 CM code) and date of visit/admission/discharge), and prescriptions or treatment (including test items). In order to protect privacy, each dataset was linkable to another dataset by a scrambled identification number (patients and hospital ID), and regulated by the Health and Welfare Data Science Centre, Ministry of Health and Welfare, Taiwan. There were a total of 1,990,322 boys (aged 0–11 years old) and 1,676,007 girls (aged 0–10 years old) in 2000, as well as 1,291,893 boys and 1,067,704 girls in 2013 (Supplementary Table S1).

The diagnosis of PP is defined as: (1) the ages at which secondary sexual characteristic development begin, if the girl is less than 8 years old and the boy is less than 9 years old; (2) accelerated bone age at least two years ahead of the age [1,2,3,4].

### 2.2. Estimation of Prevalence

We identified the ambulatory visits of newly diagnosed sexual precocity according to the International Classification of Diseases, Ninth Revision, Clinical Modification (ICD-9-CM), (ICD-9 CM code: 259.1) in boys aged 0–11 years and girls aged 0–10 years from 2000 to 2013. Precocious puberty is defined in relation to secondary sexual characteristics. The annual prevalent cases of PP were confined to ICD-9 259.1 between January and December in every calendar year. When the prevalence rate was estimated, the annual prevalent number of PP was counted and taken as the numerator, and the annual population number was cumulated from the dataset of the registry for beneficiaries. The prevalence rate was stratified by sex and age. We divided the cases into precocious puberty (PP, boys <9 and girls <8) and early puberty (EP, boys 9–11 and girls 8–10).

In general, puberty starts, on average, in girls between ages 8 and 13 years and in boys between ages 9 and 14 years [1,2,3,4]. We retrieved the Taiwan Health insurance claim database and explored the age distribution when the diagnosis was precocious puberty (ICD-9 259.1). In almost all cases, parents taking their children to the pediatric endocrinology outpatient clinic (OPD) was likely due to increased awareness of some problems such as premature adrenarche, early breast development, or the rapid progression of growth acceleration [19]. Previous studies indicated that girls aged 8–10 years and boys aged 9–11 years with secondary sexual characteristics could be considered as having reached early puberty (EP) [31,32,33].

### 2.3. Estimation of Incidence

The annual incidence of cases was identified using the first date of confirmed diagnosis. However, the first date of visit was not clear in the early period; we consider only the incidence cases after 2002. For the denominator of the incidence rate (new case/person-years) was counted annually (after excluding the deceased children or the prevalent cases before the calendar year).

In clinical practice, children were brought to the hospital when their parents noticed the symptoms of puberty. On the first visit, the pediatrician might make an inquiry and arrange a serial test to check whether PP is suspected. Generally, a true case of PP requires additional visits (including test, report, and treatment) to confirm or manage the disease. Therefore, we also identified the visit frequency of PP after the first visit, and calculated the incidence rate according to the different numbers of visit times (at least one or two visits) for PP within 1 year of diagnosis. The incidence rate was also stratified by sex and age.

### 2.4. Statistical Analysis

All analyses were performed separately by sex. The age-stratified (precocious puberty, boys <9 and girls <8; and early puberty, boys 9–11 and girls 8–10) prevalence and incidence rates were computed to explore the rates in the specific definition of puberty. Trends in the prevalence or incidence rates were estimated using join-point regression models [34,35]. The “join-point” was determined according to the Bayesian information criterion. If a join-point was detected, this indicated that the trend was modified on this time point. The annual change percentage and 95% confidence interval of rates were also estimated by join-point regression. The proportion of laboratory tests utilized within 1 year of the date of first diagnosis was calculated in confirmed cases in 2002–2007 and 2008–2012, and the differences between these two periods were compared by chi-square test. The two-sided *p* value <0.05 was considered to be statistically significant. All statistical analyses were performed by using SAS software (version 9.4; SAS Institute, Cary, NC, USA).

### 2.5. Ethical Committee Approval

The study was approved by the Institutional Review Board of Chung-Shan Medical University Hospital (protocol no: 17014. date: 8 March 2017).

## 3. Results

### 3.1. Prevalence of Precocious Puberty in Taiwan from 2000 to 2013

**Figure 1:**
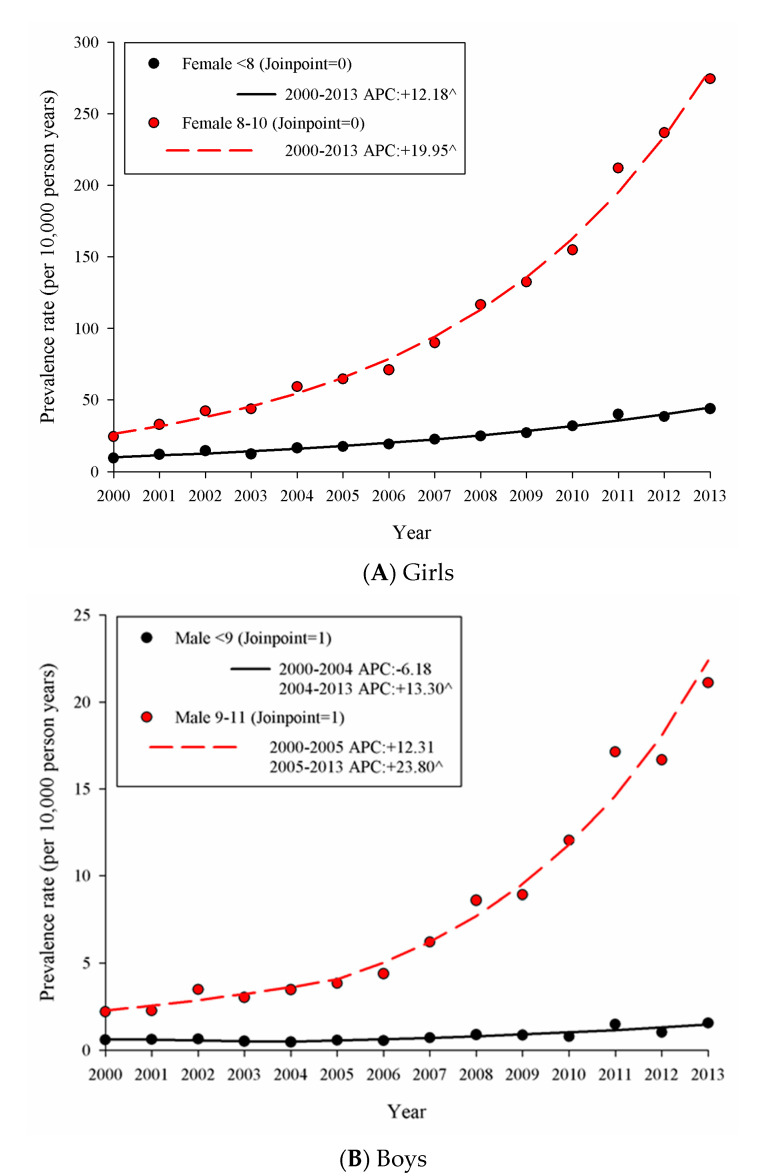
The trend of the prevalence rate of precocious puberty in (**A**) girls and (**B**) boys from 2000 to 2013; the symbol “^” indicates where the annual percent change (APC) was significantly different from zero.

In 2000, 198 boys and 2272 girls had at least one visit for PP, the crude prevalence rates (per 10,000 persons) were 0.99 (95% confidence interval [CI] 0.87–1.14) and 13.56 (95% CI 13.01–14.13) in boys and girls, respectively. In 2013, the crude prevalence rates increased to 7.01 (95% CI 6.56–7.48) and 110.95 (95% CI 108.97–112.96) in boys and girls, respectively. The sex ratio (F:M) was 13.63 (95% CI 11.78–15.76) in 2000 and 15.84 (95% CI 14.80–16.95) in 2013; the sex ratio was not significantly different between 2000 and 2013. The age-stratified trend of prevalence rate (per 10,000 persons) from 2000 to 2013 is shown in Figure 1.

For girls, there was no join-point for the 0–7 and 8–10 year-old populations. In girls aged 0–7 years, the model estimated prevalence rate of PP was 10.05 in 2000, and the significant annual percent change (APC) was +12.18%, as the rate went up to 44.79 in 2013. In girls aged 8–10 years, the estimated prevalence rates of early puberty were 26.38 and 280.85 in 2000 and 2013, respectively, with the significant APC at +19.95%. For boys, there was one join-point for the prevalence rate in each age group (the join-point was 2004 and 2005 in ages 0–8 and 9–11 years, respectively). In boys aged 0–8 years, there was no significant APC from 2000 to 2004; nevertheless, the APC (+13.30%) was significant from 2004 to 2013, and the prevalence rates were 0.63, 0.48, and 1.47 in 2000, 2004, and 2013, respectively. In boys aged 9–11 years, a significant APC (+23.80%) was observed from 2005 to 2013, and the prevalence rates were 2.27, 4.06, and 22.39 in 2000, 2004, and 2013, respectively.

### 3.2. Incidence of Precocious Puberty in Taiwan from 2002 to 2013

**Figure 2:**
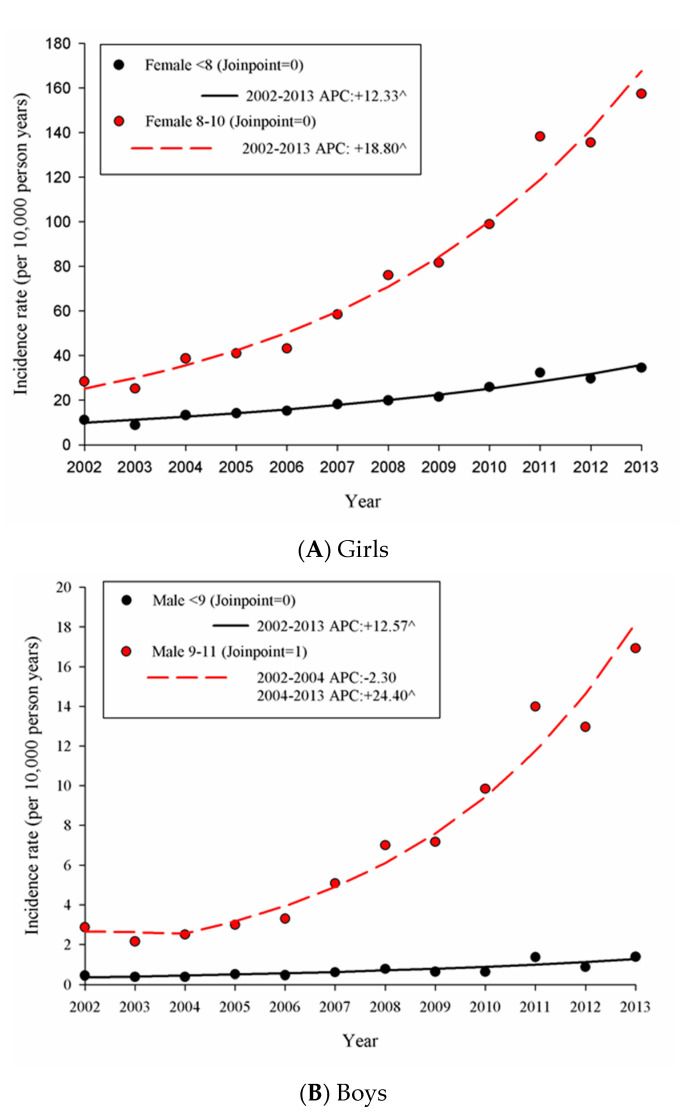
The trend of the PP incidence rate in (**A**) girls and (**B**) boys from 2002 to 2013, where the symbol “^” indicates that the annual percent change (APC) was significantly different from zero.

For girls aged 0–10 years, a total of 2584, and 7498 incident cases (≥1 outpatient visit) of PP were identified. The incidence rates (per 10,000 person years) were 16.17 (95% CI 15.55–16.80) and 70.23 (95% CI 68.65–71.83) in 2002 and 2013, respectively. For boys aged 0–11 years, a total of 207 and 739 incident cases were identified in 2002 and 2013, and the incidence rates were 1.09 (95% CI 0.95–1.24) and 5.72 (95% CI 5.32–6.15), respectively. The sex ratio (F:M) was 14.89 (95% CI 12.92–17.16) in 2002 and 12.28 (95% CI 11.38–13.24) in 2013. The trend of the incidence rate (per 10,000 person-years) from 2002 to 2013 is shown in Figure 2.

**Figure 3:**
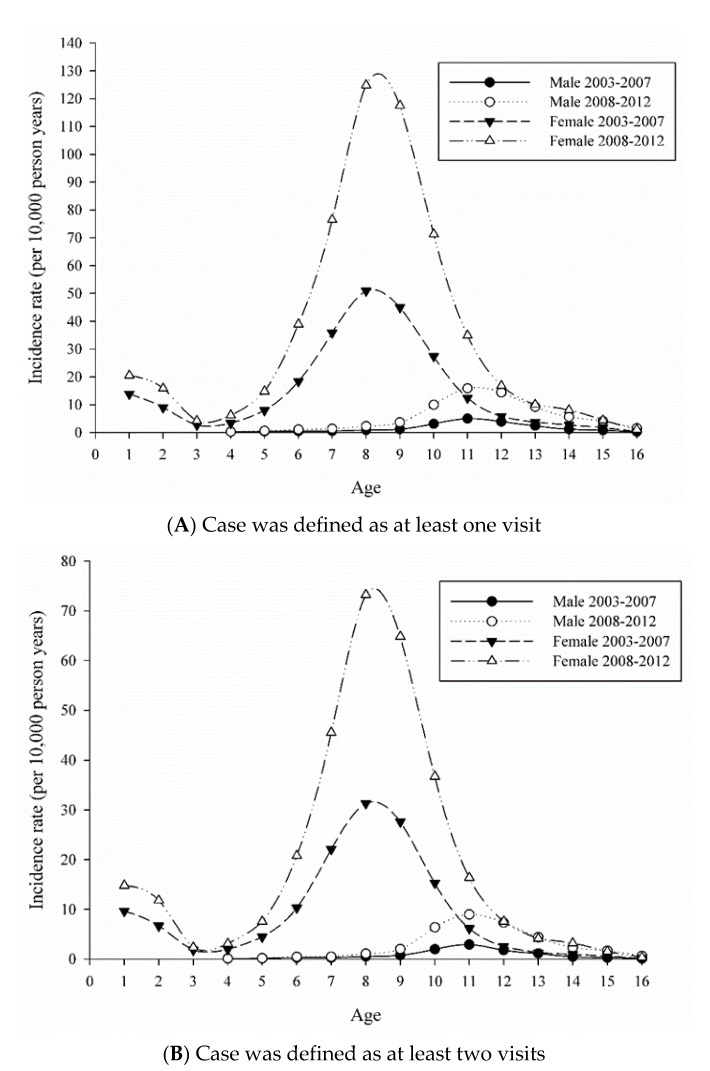
The age-specific incidence of PP in Taiwan by outpatient visit within 1 year from the first diagnosis. (**A**) at least one visit and (**B**) at least two visits.

In girls aged 0–7 years, the APC (+12.33%) of the incidence rate was significant from 2002 to 2013, with incidence rates of 9.95 and 35.77 in 2002 and 2013, respectively. In girls aged 8–10 years, the APC (+18.80%) was significant from 2002 to 2013, with incidence rates of 25.22 and 167.75 in 2002 and 2013, respectively. For boys aged 0–8 years, the APC was +12.57% and was significant from 2002 to 2013, and the incidence rates were 0.34 and 1.26 in 2002 and 2013, respectively. For boys aged 9–11 years, the APC was +24.40% and was significant from 2004 to 2013, and the incidence rates were 2.67, 2.55, and 18.18 in 2002, 2004, and 2013, respectively. Figure 3 showed the age-specific incidence of PP during 2003–2007 and 2008–2012.

The sensitivity analysis was conducted when the incidence cases were defined by the number of outpatient visits within 1 year from the diagnosis. The age-specific incidence rate demonstrated a bimodal distribution in girls; the first peak was found before 2 years old, and another peak was observed at 6–10 years. On the other hand, the one peak distribution was estimated in boys; this peak was at 9–13 years. Figure 3B shows that the incidence was half of Figure 3A, indicating that only 1 in 2 patients required advanced care after the first visit.

## 4. Discussion

To our knowledge, this is the first register-based (NHIRD), nationwide, epidemiologic study on PP development in Taiwan. Both the incidence (2002–2013) and prevalence (2000–2013) of PP (age 0–8 years in girls; age 0–9 years and in boys) and early precocious incidence (age 8–11 years in girls; 9–12 years in boys) were found to be increasing, which is similar to other previous studies [26,27,28,29]. In addition, we observed a higher rate of outpatient visits for PP after 2004, which might have been associated with the Chinese Milk Formula Incident (the melamine adulteration in milk powder) in 2008 [36] and the Taiwan Food Incident (the plasticizer DEHP contained in food and drinks as a clouding agent.) in 2011 [24,25]. Following these two events, the number of PP outpatient visits increased; however, these incidents may not bear any direct relation to PP. Long-term exposure to environmental hormones has been proven to cause PP [5,19,20,21,22]. We considered that the incidents might have alerted parents’ attention to food safety issues and children’s health status, and additionally, Taiwan NIH provides free and convenient access to healthcare with very modest co-payment by most clinics and hospitals [30].

Teilmann et al. conducted a nationwide register-based epidemiologic study in Denmark. Cases were identified with an International Statistical Classification of Diseases 0th Revision (ICD-10) diagnosis (E30.1 or E22.8) [29]. The diagnostic age for PP was the onset of puberty before 9 years of age for girls and 10 years for boys, with a prevalence of 20–23 per 10,000 in girls, and <5 per 10,000 in boys [29]. Teilmann et al. indicated that the general clinical definitions of PP age <8 years old in girls and <9 years in boys were not appropriate for database analysis, because there might be a time lag of up to 1 year between the first awareness of signs of puberty by the parents and the establishment of diagnosis by the pediatric endocrinologist [29]. Teilmann et al. also indicated that in their sample, including central precocious puberty (CPP), premature thelarche, and premature adrenarche, pubertal development was found early but within the normal age limits, leading to a misclassification and registration in files, which is similar to our results [29]. In a Spanish study, the annual incidence rate of girls with CPP was estimated to be only between 0.013 and 0.217 per 10,000 (boys, 0 and 0.023), and the prevalence was 0.19 per 10,000 in girls (boys, 0.46) [28]. A Korean study reported that the overall prevalence of CPP in 2010 was 2.61 per 10,000 children, with a prevalence of 5.59 per 10,000 girls and 0.17 per 10,000 boys [27].

Differences from the definition of cases, research methods, and race may affect the analysis results. In this study period, we demonstrated that the prevalence (per 10,000) was 9.37 in 2000 and increased to 43.92 in 2013 in girls <8 years. On the other hand, the prevalence was 0.58 in 2000 and increased to 1.54 in 2013 in boys <9 years old, which is higher than certain previous reports [26,27,28,29]. Our study may have a higher estimation of prevalence; however, if a more rigorous definition was used it could lead to the neglect of children’s developmental problems, such as premature thelarche, premature adrenarche, and pubertal development, which were found early within the normal age for long-term outpatient follow up. Practically, many parents were willing to self-pay for GnRHa treatment for their children who did not meet the payment requirements of the National Health Insurance. This study, therefore, may not be directly comparable to other studies based on the individuality of national culture, race, and different study design. On the other hand, we consider that environmental pollution issues may have a long-term effect on children’s health in Taiwan.

The population-based NHIRD, maintained by the National Health Research Institutes, is derived from the claims data of the National Health Insurance program [30]. This database originates from the administrative databases whose main function is for imbursement [30,35,37]. During the period of 2000–2013, the diagnostic codes, such as the ICD9-CM codes, were commonly used to identify PP patients. The National Insurance Health Administration requested all clinics or hospitals to use the ICD-9 code as the diagnosis code [37]. The ICD-9-CM code of 259.1 was accordingly used for the diagnosis of precocious sexual development and puberty, but not meticulously classified elsewhere. The ICD-9-CM code, however, could not identify premature thelarche, premature adrenarche, and the pubertal development status.

The NIHRD database analyses with the ICD9-CM codes, which included patients with true CPP, premature thelarche, premature adrenarche, and delays in seeking medical attention for precocious puberty, might have led to an overestimation of PP. A limitation of this current study must be considered because this out of date database could not, as aforementioned, identify premature thelarche, premature adrenarche, and pubertal development status. We were unable to calculate, exclude, or differentiate these patients accurately from this study.

Since 2015, the ICD-10 codes have been used to define the diagnoses by the Taiwan National Health Insurance Administration. The ICD-10-CM has identified more diagnostic classifications, such as diagnosis ICD-10-CM Code E30.8: Other disorders of puberty; ICD-10-CM Diagnosis Code E30.1: Precocious puberty; ICD-10-CM Diagnosis Code E27.0: Other adrenocortical over activity [38]. We can accurately calculate, exclude, and differentiate patients with the ICD-10 classifications in future studies.

Le Moal et al. used the national insurance database (France, 2011–2013, ICD 10 codes) to select all cases for which at least one reimbursement had been paid for gonadotrophin-releasing hormone (GnRH) agonists, and to exclude cases for which a cause of CPP had been identified (e.g., surgical intervention for brain tumors or other brain-related causes) and peripheral endocrine tumors [26]. They reported a national annual incidence of 2.68 (95% CI: 2.55, 2.81) per 10,000 girls under the age of 9 years and 0.24 (95% CI: 0.21, 0.27) per 10,000 boys under the age of 10 years. In Korea, the overall incidence of CPP with treatment was 1.53/10,000 girls (<8 years) and 0.06/10,000 boys [27]. Soriano-Guillén L et al. estimated that the global incidence rate of CPP with GnRH agonists treatment was 5.66 cases per million person-years at risk, with an annual incidence ranging between 0.002 and 0.107 per 10,000 [28]. The Denmark annual incidence of PP is 0.5 per 10,000 in girls younger than 2 years, decreasing to levels below 0.05 per 10,000 in girls aged 2 to 4 years, 8 per 10,000 for girls aged 5 to 9 years, and 1 to 2 per 10,000 in boys aged 8 to 10 years [29]. In our study, from 2000 to 2013, the number of visits to pediatric endocrinology outpatient clinics for precocious puberty increased for both genders, which is similar to previous studies [26,27,28,29].

We found that certain children were diagnosed with PP for girls ≥11 years old and boys ≥12 years old using NHIRD. From this result, it is interesting that boys aged 12–18 years and girls aged 11–18 years had a higher outpatient visit rate for pp. There might be two reasons for this; first, these children in 2003–2007 might have had a delay in seeking medical attention due to the parents’ lack of knowledge regarding puberty development [39,40,41,42]; secondly, Taiwan inhabitants and enrollees enjoy almost free access to healthcare with very modest co-payments at most clinics and hospitals, which encouraged the parents to take their children to outpatient visits.

In this study, the number of children’s outpatient visits for PP increased since 2008, which might have been due to the Chinese Milk Formula Incident (melamine adulteration in milk powder) in 2008 [36], leading to an estimation that about 1/2 of the patients required advanced care (Figure 3). When the medical tests were further analyzed, there were approximately 90% of patients who received bone-age examination, 50–60% who required luteinizing hormone (LH) and follicle-stimulating hormone (FSH) surveys, and 22–31% who required a luteinising-hormone releasing hormone (LHRH) stimulation test (Supplementary Tables S2-1, and S2-2). However, half of the bone-age tests returned normal, whereas only 50% of patients required advanced evaluation and follow-up. In Taiwan, awareness of how to recognize and define health problems could affect patient help-seeking behavior. We speculate that the parents received information regarding PP, perhaps from friends or the media, and they felt worried or uncertain about their children’s health status. From the results of this study, it seems to be implied that parents may have excessive anxiety or a lack of cognition regarding their children’s pubertal development or health status. We could explore this issue in the future and help to decrease the waste of medical resources.

Previous studies reported that certain girls with non-progressive or intermittently progressive PP might have rapidly progressed into CPP, which requires further examinations and medical treatment [43,44,45]. In Taiwan, the National Health Insurance provides reimbursement of GnRH analogue treatment in children with rapidly progressed CPP.

## 5. Study Limitations

There were certain limitations of this study in terms of the analytic findings when the NHIRD was used. First, the accuracy and completeness of the ICD-9 coding used by the NHIRD was untested. According to the ICD-9 coding, we were unable to distinguish between premature thelarche and premature adrenarche, and the advanced bone age might lead to an overestimation of PP. Secondly, in Taiwan, the outpatient service was covered by the National Health Insurance (NHI) copayment, which might lead to an overuse of medical resources.

## 6. Conclusions

Precocious puberty is currently a medically concerning problem in developed and developing countries. In this population-based study from 2000 to 2013, the number of visits to pediatric endocrinology outpatient clinics for precocious puberty increased for both genders. Although the epidemiology of PP in this study was limited due to the administrative data of NHIRD, we should encourage future studies of precocious puberty. We advocate that paying increased attention to children’s healthy, environmental hormones and diet is important, as precocious puberty may increase the physiological and psychological problems in children.

## Supplementary Materials

The following are available online at https://www.mdpi.com/1660-4601/17/18/6765/s1, Table S1: Population number in this study, Table S2-1: Received tests within 1 year from diagnosis of precocious puberty in girls <8 years old, boys <9 years old, Table S2-2: Received tests within 1 year from diagnosis of precocious puberty in girls 8–10 years old, boys 9–11 years old.
