# Supplementary material for: The Age Distribution among Children Seeking Medical Treatment for Precocious Puberty in Taiwan
Source: Int J Environ Res Public Health. 2020 Sep 17;17(18):6765. doi: 10.3390/ijerph17186765 (PMC7559721; doi:10.3390/ijerph17186765)
Supplement: Supplementary file 1 [file ijerph-17-06765-s001.pdf]

Supplementary Table 1. Population number in this study

	Male		Female	
Year	Aged 0 to <9	Aged 9 to 11	Aged 0 to <8	Aged 8 to 10
2000	1,481,361	508,961	1,207,338	468,669
2001	1,447,358	515,560	1,173,031	466,951
2002	1,397,659	509,049	1,128,722	469,785
2003	1,347,068	510,734	1,078,881	474,036
2004	1,291,477	512,661	1,026,855	471,906
2005	1,230,669	511,406	970,698	471,843
2006	1,169,195	511,446	940,358	442,781
2007	1,132,503	478,524	899,308	420,676
2008	1,086,728	457,268	846,099	411,240
2009	1,026,193	447,637	814,604	405,142
2010	978,216	441,205	776,023	386,472
2011	950,914	421,249	761,852	347,564
2012	952,310	380,406	770,473	328,106
2013	930,767	361,126	757,304	310,400

Supplementary Table 2-1. Received tests within 1 year from diagnosis of precocious puberty in girls <8 years old, boys <9 years old

	Proportion (%) among incident cases					
	Male aged <9 years old			Female aged<8 years old		
Exam item	2003-2007	2008-2012	p value	2003-2007	2008-2012	p value
Bone age	71.63	83.37	0.0857	86.30	87.33	0.4801
Testosterone	45.39	47.98	0.6232	5.90	4.23	<.0001
E2[a]	28.72	28.74	0.9966	58.50	60.32	0.1324
LH[b]	45.74	45.13	0.9057	57.87	59.57	0.1580
FSH[c]	45.39	38.95	0.1949	55.87	57.28	0.2335
LHRH stimulating test[d]	16.67	10.93	0.0418	18.27	15.35	<.0001
Progesterone	22.70	10.93	0.0002	7.12	2.82	<.0001
Thyroid function	29.08	35.63	0.1390	15.52	21.62	<.0001
Insulin and diabetes related tests	15.96	26.37	0.0045	5.52	9.85	<.0001
HCG[e]	22.70	23.28	0.8746	22.67	18.35	<.0001
Prolactin	20.21	17.58	0.4279	27.14	26.02	0.1681
Ultrasonography	38.30	30.40	0.0773	57.98	61.42	0.0047
Skull X-ray	19.86	26.60	0.0740	14.35	17.34	<.0001
MRI[f]	10.64	4.28	0.0022	5.83	6.21	0.3264

a. Estradiol, E2; b. Luteinizing hormone, LH;c. follicle-stimulating hormone, FSH;d. Luteinizing-hormone releasing hormone, LHRH; e. human chorionic gonadotropin, HCG;f. Magnetic Resonance Imaging, MRI.

Supplementary Table 2-2. Received tests within 1 year from diagnosis of precocious puberty in girls 8-10 years old, boys 9-11 years old

	Proportion (%) among incident cases					
	Male aged 9-11 years old			Female aged 8-10 years old		
Exam item	2003-2007	2008-2012	p value	2003-2007	2008-2012	p value
Bone age	88.17	93.42	0.1857	90.57	92.66	0.0838
Testosterone	69.24	69.03	0.9514	8.85	6.57	<.0001
E2[a]	32.63	33.33	0.7668	61.96	62.17	0.8311
LH[b]	69.86	68.20	0.6266	61.56	61.02	0.5837
FSH[c]	68.87	62.59	0.0582	59.64	57.58	0.0321
LHRH stimulating test[d]	31.38	26.01	0.0133	26.75	22.78	<.0001
Progesterone	29.27	21.60	0.0001	6.96	4.89	<.0001
Thyroid function	40.22	45.25	0.0667	20.71	30.53	<.0001
Insulin and diabetes related tests	19.68	32.92	<.0001	10.62	19.53	<.0001
HCG[e]	30.51	40.15	0.0001	22.82	21.54	0.0299
Prolactin	25.65	30.18	0.0421	28.33	28.66	0.6231
Ultrasonography	24.41	29.76	0.0151	55.43	56.97	0.1033
Skull X-ray	20.42	36.16	<.0001	17.82	25.16	<.0001
MRI[f]	19.80	15.25	0.0069	9.57	7.38	<.0001

a. Estradiol, E2; b. Luteinizing hormone, LH;c. follicle-stimulating hormone, FSH;d. Luteinizing-hormone releasing hormone, LHRH; e. human chorionic gonadotropin, HCG;f. Magnetic Resonance Imaging, MRI.
